# Kidney Retransplantation after Graft Failure: Variables Influencing Long-Term Survival

**DOI:** 10.1155/2022/3397751

**Published:** 2022-06-22

**Authors:** Jonas Ehrsam, Fabian Rössler, Karoline Horisberger, Kerstin Hübel, Jakob Nilsson, Olivier de Rougemont

**Affiliations:** ^1^Department of Thoracic Surgery, University Hospital Zurich, Zürich, Switzerland; ^2^Department of Surgery and Transplantation, University Hospital Zurich, Zürich, Switzerland; ^3^Department of Nephrology, University Hospital Zurich, Zürich, Switzerland; ^4^Department of Immunology, University Hospital Zurich, Zürich, Switzerland

## Abstract

**Background:**

There is an increasing demand for kidney retransplantation. Most studies report inferior outcomes compared to primary transplantation, consequently feeding an ethical dilemma in the context of chronic organ shortage.

**Objective:**

To assess variables influencing long-term graft survival after kidney retransplantation. *Material and Methods*. All patients transplanted at our center between 2000 and 2016 were analyzed retrospectively. Survival was estimated with the Kaplan–Meier method, and risk factors were identified using multiple Cox regression.

**Results:**

We performed 1,376 primary kidney transplantations and 222 retransplantations. The rate of retransplantation was 67.8% after the first graft loss, with a comparable 10-year graft survival compared to primary transplantation (67% vs. 64%, *p*=0.104) but an inferior graft survival thereafter (log-rank *p*=0.026). Independent risk factors for graft survival in retransplantation were age ≥ 50 years, time on dialysis ≥1 year, previous graft survival <2 years, ≥1 mild comorbidity in the Charlson–Deyo index, active smoking, and life-threatening complications (Clavien–Dindo grade IV) at first transplantation.

**Conclusion:**

Graft survival is comparable for first and second kidney transplantation within the first 10 years. Risk factors for poor outcomes after retransplantation are previous graft survival, dialysis time after graft failure, recipient age, comorbidities, and smoking. Patients with transplant failure should have access to retransplantation as early as possible.

## 1. Introduction

The expansion of indications for kidney transplantation and the increasing use of extended criteria grafts inevitably lead to a higher number of patients with graft failure. Given the current high overall rate of kidney transplants, which will assumably continue to rise, the growing number of patients requiring kidney retransplantation is a challenge [1]. Furthermore, recent changes in organ allocation policy, with higher allocation scores for human leukocyte antigen (HLA) antibodies, indirectly enhance the likelihood of retransplantation in patients after graft failure [2, 3]. Patients undergoing kidney retransplantation benefit from improved survival [4–6] and better quality of life [7] compared to patients who remain on dialysis in this situation. In fact, return to dialysis after graft failure has poor outcomes with high mortality rates [8–11]. The outcome is then even inferior to dialysis prior to first transplantation [12]. Graft survival data after retransplantation are, however, controversial. Some studies report inferior outcomes compared to primary transplantation [13–15], while others present comparable results for retransplantations in the long-term follow-up [1, 16]. Data on risk factors for poor outcomes after retransplantation though are scarce. In the present work, as a first step, we characterize the cohort of patients who have received a second kidney transplant. The following and actual aim of the work is to identify candidate profiles who will benefit from retransplantation.

## 2. Methods

We retrospectively reviewed medical records of all patients who underwent kidney transplantation at our institution between January 1, 2000, and December 31, 2016. Patients were followed up until December 31, 2017. Due to the low number of third and fourth transplantations, we assessed first and second kidney transplantations only. The local ethics committee reviewed and approved the study protocol (project number 2018–00153).

We assessed recipient characteristics, including pre-, intra-, and postoperative parameters. The age-independent Charlson–Deyo index was used to estimate the impact of multiple recipient comorbidities [17]. Graft survival was defined as the time from transplantation until the patient returned to dialysis or died with a functioning graft. Patient survival was defined as the time from transplantation to death of the patient with or without a functional graft. Death-censored graft survival was defined by the time from transplantation to return to dialysis while censoring death with a functional graft. Extended criteria donor (ECD) was defined as age ≥60 years or 50 to 59 years with at least 2 of the following: arterial hypertension, serum creatinine level 132 *μ*mol/l, and cerebrovascular cause of death [18].

Human leukocyte antigen (HLA) mismatch was defined as the number of mismatches within HLA A, B, and DR, totaling a maximum of 6 possible mismatches. HLA-donor-specific antibodies (DSA) detection was using a sensitive single antigen bead assay (Luminex®), and intensity was measured by mean fluorescence intensity (MFI). MFI levels above 1,000 were considered relevant. No patients were excluded from analysis, irrespective of MFI levels or previous episodes of acute rejection. However, it was our policy to avoid transplantations with DSA, especially with MFI levels > 1,000, whenever possible. All patients that underwent retransplantation received monoclonal antibodies for induction therapy (Thymoglobulin® or Basiliximab®).

We accounted our models for era effects, which occurred in May 2008 due to the start of allocation according to donor-specific antibodies. Complications were assessed according to the validated and severity-oriented Clavien–Dindo classification [19, 20].

### 2.1. Statistical Analysis

Statistical analysis was performed with IBM SPSS Statistics 25.0 (IBM Corp, 2015, Armonk, NY) and Stata 13.1 (StataCorp LP, College Station, Tex). Categorical variables were compared by chi-square test or Fisher exact test for expected frequencies <5. Continuous variables were analyzed by the Mann–Whitney test. Survival was estimated by the Kaplan–Meier method and compared with the log-rank test. Cox regression was used to assess risk factors for graft failure (using the time to failure or death as a dependent variable) as well as the chance of retransplantation (using the time to retransplantation as a dependent variable). First, every variable was checked with a univariate (enter) model. Variables with a *p* value <0.2 [21] were tested in a multivariable stepwise backward Cox regression model. The number of factors introduced into the final multivariable models was calculated by considering sample size and the number of occurring events (graft failure and retransplantation, respectively) [22]. To confirm that variables show a stable significance, they had to be frequent in number. Linear regression was used to test collinearity between variables. A variance inflation factor >5 and a tolerance <0.2 was defined as indicating a collinearity problem.

## 3. Results

### 3.1. Donor and Recipient Characteristics

Out of 1,598 kidney transplantations, 1,376 (86.1%) were first transplantations, 192 (12.0%) second, 28 (1.8%) third, and 2 (0.1%) were fourth transplantations. Loss of follow-up after first and second transplantations was 3.1% (*n* = 43) and 1.6% (*n* = 3), respectively. Second transplant patients had less diabetes or cystic disease as the underlying cause of primary kidney failure compared to first transplantation, 15.9% versus 6.8% and 16.7% versus 10.4%, respectively. The median duration of dialysis between first and second transplantation was longer than before the first transplant, 2.5 versus 1.7 years, respectively. Second transplant recipients were less likely to get an ECD or donor after circulatory death (DCD) graft, 25.9% versus 6.3% and 6.8% versus 0%, respectively. The living donation was also less frequent in the setting of second transplantation (32.3% vs. 22.4%). Detailed recipient characteristics are listed in Table 1.

Within our study period, 283 patients were evaluated for a first retransplantation. Of these 192 (67.8%) underwent a retransplantation. The main reason for the decline in patients for retransplantation was reduced physical condition, followed by malignancies and infection (Table 2).

### 3.2. Graft and Patient Survival

**Figure 1:**
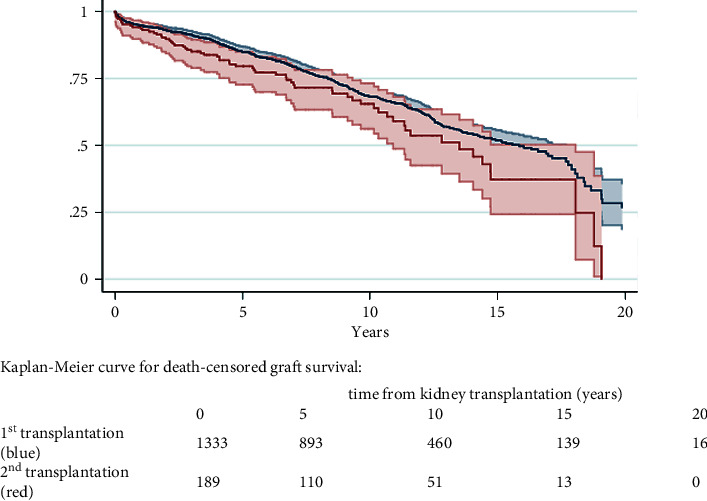
Graft survival.

Graft survival is shown in Figure 1. Median graft survival for first and second kidney transplantation was 15.3 (95% CI 13.9–16.8) and 13.5 (95% CI 11.0–16.0) years, respectively. Overall graft loss was 35.1% and 36.0% after first and second kidney transplantation, respectively.

While no significant difference in graft survival was seen within the first 10 years after transplantation (*p*=0.104), this changed over time and was then lower for retransplantation (*p*=0.026). Multiple Cox regression model, adjusted for recipient, donor, and era variables, identified the second transplantation as a risk factor for lower graft survival (*p*=0.003; Table 3).

The mortality rate was 16.8% and 16.9% after the first and second transplantation, respectively (*p*=0.9). Causes of death between the two groups did not differ significantly, with infections, heart failure, and malignancies being the main reasons for death in both groups.

### 3.3. Likelihood and Risk Factors for Retransplantation

Using time from graft loss to retransplantation as a dependent variable in a multiple Cox regression model, chances of retransplantation were higher among candidates younger than 65 years at the time of first graft loss, with less comorbidities (Charlson–Deyo index ≤ 3), and less than 3 years on dialysis. Previous graft survival of more than 5 years was also a positive prognostic factor for being retransplanted (Table 4). Multiple Cox regression model (Table 5) revealed the following as risk factors for graft survival in retransplantation: age ≥ 50 years, Charlson–Deyo index ≥ 3 (equal to ≥ 1 mild comorbidity), time on dialysis ≥ 1 year, previous graft survival < 2 years, active smoking, and life-threatening complications (Clavien–Dindo grade IV) at first transplantation. Previous graft loss due to acute rejection, a number of HLA mismatches, and DSA did not have a significant impact on the loss of the subsequent graft. When the detected risk factors for retransplantation (Table 5) were compared to the risk factors in the overall population (Table 3), retransplantation was revealed to be more vulnerable in regard to increasing age, comorbidities, and length of the previous dialysis, but not for increasing HLA mismatch.

## 4. Discussion

Kidney retransplantation accounts for approximately 15% of transplant activity [14, 23, 24], a number constantly increasing over the past two decades. Within this cohort, nearly two-thirds of patients losing their first graft were retransplanted. In times of chronic organ shortage, growing rates of retransplantations fuel the delicate discussion on utility and fairness.

Reports on graft survival after kidney retransplantation are conflicting and the prognosis of retransplantation compared to first transplantation remains unclear. Within our cohort, graft and patient survival rates within 10 years were comparable for first and second transplantations and seem to decline thereafter, favoring first transplantation. This time lag difference in outcome has been demonstrated before. Trèbern-Launay et al. showed an increased risk for graft failure in retransplantations, with a delayed effect becoming evident beyond 4 years of follow-up [13]. Magee et al. 2007 reported worse outcomes in retransplantation already from the first year post-transplantation [14]. On the other hand, there is data reporting comparable graft survival rates for retransplantation and first transplantation [16, 25]. Arnol et al. reported almost identical long-term graft survival up to 10 and 15 years [16]. Of note, in this particular study, the time between first and second transplantations was exceptionally short, with a median dialysis time of only 16 months. Another series reported equal incidence rates of acute rejection and 8-year survival rates [1].

There are currently no recommendations for the management of patients with a failed kidney transplant, nor are there clear decision criteria for relisting. To date, no scoring system to predict outcome after repeat kidney transplantation, such as the lung allocation score for lung transplantation [26], is available. Published data on this subject essentially has in common that the results of retransplantation are influenced by the outcome of the first transplantation [16, 27–29]. This is consistent with our analysis in which prolonged primary graft survival was protective for retransplant outcome. Nevertheless, recommendations for the management of patients after graft failure cannot yet be derived from this.

Our analyses show that there are indeed some risk factors for second graft loss and should have an impact on the management of patients after graft loss at initial transplantation. In our cohort, dialysis duration before retransplantation is a considerably stronger risk factor for graft loss than before initial transplantation. While dialysis for 2 or more years was a risk factor for first graft failure, the risk of subsequent organ loss after retransplantation is already present after 1 year of dialysis. These data are consistent with reports on the negative effect of waiting time and dialysis on kidney transplant outcomes [30–32], but the impact appears to be compounded after a second transplant. Dialysis time of less than 3 years significantly increased the likelihood of retransplantation in our cohort. Hence, the duration of dialysis is one of the strongest influencing factors for retransplantation. The patients in our cohort were on dialysis for an average of over 600 days before being retransplanted. This time on dialysis before repeat transplantation was long compared to other reports [16], probably reflecting low access to living donation for retransplantation. Results after living donor retransplantation are still superior compared to primary deceased donor transplantation [14], and preemptive living donations have a well-known beneficial effect on long-term graft survival, minimizing the morbidity associated with dialysis restart and improving clinical outcomes [30]. In our cohort, however, we did not detect a direct benefit for preemptive retransplantations, but we detected that duration of dialysis before retransplantation was a stronger risk factor for graft loss than the duration of dialysis before initial transplantation. This is particularly evident when dialysis time is exceeding one year. This indirectly suggests a beneficial effect for preemptive retransplantations.

Of note, smoking was the strongest risk factor for graft loss after retransplantation in our cohort. The adverse effects of smoking have been reported for primary transplants, being associated with an increased risk of graft loss and death [33, 34]. However, this has not yet been described for retransplantation. Consistent with prior studies, recipient age was a risk factor for graft loss [35, 36]. While the risk of graft loss for first transplants increased from the age of 60, this is true for retransplants already from 50. Interestingly, severe obesity was not a risk factor for retransplantation in our cohort. This is in contrast to previous studies [13, 28] and the general knowledge of elevated surgical risks in obese recipients [37–39]. However, this missing effect was probably related to the overall low rate of obese recipients in our cohort.

Retransplantations carry an increased immunological risk regarding rejection and complications of immunosuppression [16, 40]. However, in our cohort, a number of HLA mismatches were not a risk factor for graft survival, even though the number was higher for retransplantations. This is in line with previous studies [28, 41] but conversely to others [42]. The same is true for DSA. While the total number of DSA and rates of MFI were higher in retransplantations, we did not detect any influence on graft survival. In addition, we did not detect acute rejection as a predictor of second graft survival. This is conflicting with previous results that identified preformed DSA as a risk factor for acute rejection and graft loss in first transplantation and retransplantations [43, 44]. This missing association between immunologic risk factors and long-term retransplant outcome in our cohort has likely been positively influenced by our strict attempts to avoid DSA and MFI levels >1,000 in the last decade.

This study has some limitations. It is a retrospective analysis and readers need to consider the limitations and error rate of data. Loss of follow-up was very low for the transplanted population. However, we did not compare the results to a matched dialysis cohort after graft loss. Limited statement regarding survival benefits of retransplantation compared to staying on dialysis is possible.

Our results highlight the importance of including prior transplant outcomes to improve decision-making for repeat wait-listing and predicting retransplant outcomes. As long as consensus on relisting is lacking, a clear definition of risk factors is crucial to optimize patient selection for repeated transplantation. With this long-monitored cohort of retransplanted patients, we were able to identify risk factors that increase the risk of graft loss and should therefore be taken into account in the preparation for retransplantation. Most importantly, the data show that early relisting, which is associated with younger age of the patient and shorter dialysis time, increases the chances of longer graft survival. Relisting should therefore be initiated promptly, potentially before graft failure. However, this might be limited in patients with primary disease recurrence-related graft failure or those who lost their graft for antibody-mediated rejection. In this regard, the data also clearly show that patient adherence is essential medically but also with a focus on lifestyle, as a BMI <30 is more likely to lead to relisting and nicotine abstinence to longer graft survival.

## 5. Conclusion

Kidney retransplantation has comparable long-term outcomes to primary transplantation. Outcome after retransplantation depends on previous graft survival, dialysis time after first graft failure, recipient age, comorbidities, and smoking. Due to the excellent long-term outcome, patients with transplant failure should be offered retransplantation at the earliest.

## Figures and Tables

**Table 1:** Patient characteristics and surgical details.

Patient's characteristics	1st transplantation	2nd transplantation	*p* value
*n*	1,376	192	
Age, median (years, range)	48.4 (2–81)	46.2 (13–73)	0.3
Gender (male/female (%))	63.2/36.8	62.5/37.5	0.8

Time on dialysis, median (years, range) 1–3 years ≥3 years CAPD	1.7 (0–15.5) 436 (32.7%) 408 (30.6%) 218 (15.8%)	2.5 (0–17.7) 55 (29.4%) 85 (45.5%) 43 (22.4%)	<0.001 0.002 <0.001 0.02

Time on the waiting list, median (days, range)	329 (0–13,951)	623 (0–4,443)	0.1

BMI (kg/cm^2^)			
>30	138 (10.0%)	11 (5.7%)	0.06
<17	8 (0.6%)	2 (1.0%)	0.4

Underlying disease			
Diabetes	219 (15.9%)	13 (6.8%)	<0.001
Hypertension	204 (14.8%)	21 (10.9%)	0.2
Glomerulopathy	238 (17.3%)	57 (29.7%)	<0.001
Cystic disease	230 (16.7%)	20 (10.4%)	0.02

Types of transplantation			
Preemptive	232 (16.9%)	14 (7.3%)	0.001
Living donors	443 (32.3%)	149 (22.4%)	0.006
(i) Unrelated	239 (54%)	92 (62%)	
(ii) Related	204 (46%)	57 (38%)	
(1) 1^st^ degree	94%	82%	
(2) 2^nd^ degree	6%	18%	
DCD	94 (6.8%)	2 (1.0%)	0.002
Extended donors	357 (25.9%)	12 (6.3%)	<0.001

Charlson–Deyo index, median	2 (2–8)	2 (2–6)	0.6
Cold ischemia time, median (hours)	8 (1–37)	9 (1–29)	0.6

HLA mismatches			
Median (range)	3 (0–6)	4 (0–6)	<0.001
0	92 (6.7%)	2 (1.0%)	0.002
1–2	347 (25.3%)	35 (18.2%)	0.03
3–5	831 (60.5%)	136 (70.8%)	0.005
≥6	104 (7.6%)	19 (9.9%)	0.05

DSA			
Any patient with DSA	638	101	
Median number (range) of DSA	0 (0–5)	0 (0–6)	<0.001
Patients with:			
No DSA	541 (84.8%)	54 (53.5%)	<0.001
1 DSA	68 (10.7%)	29 (28.7%)	<0.001
2 DSA	20 (3.1%)	12 (11.9%)	<0.001
≥3 DSA	9 (1.4%)	6 (5.9%)	0.01

DSA cumulative MFI			
Median (range)	0 (0–12,949)	0 (0–25,741)	<0.001
Mean ± SD	361+/−50.6	2,087+/−432	
0	541 (84.8%)	54 (53.5%)	<0.001
1–1,000	38 (6.0%)	12 (11.9%)	0.03
1,001–2,000	25 (3.9%)	11 (10.9%)	0.002
>2,000	34 (5.3%)	24 (23.8%)	<0.001

Clavien–Dindo index, major			
IIIb	8.8%	8.9%	0.9
IVa	3.1%	2.6%	0.2
IVb	0.9%	0%	0.4
V	0.7%	1.0%	0.6

*Note.* BMI, body mass index (kg/m^2^); CAPD, continuous ambulatory peritoneal dialysis; DCD, donor after circulatory death; HLA, human leukocyte antigen; DSA, donor-specific antibody; MFI, mean fluorescence densitometry; SD, standard deviation.

**Table 2:** Listing for retransplantation.

	2^nd^ transplantation
*n*	283
Relisted and retransplanted	192 (67.8%)

On waiting list Death on waiting list Removed from list Patient moved to another country	22 (7.8%) 2 (0.7%) 1 (0.4%) 1 (0.4%)

Death while evaluation for wait-listing	6 (2.1%)

Excluded from retransplantation due to reduced general condition	24 (8.5%)
Malignancy	5 (1.8%)
Infection	3 (1.1%)
Increased risk for rejection	2 (0.7%
Compliance problems	3 (1.1%)
Unknown	6 (2.3%)

Patient refused relisting	15 (5.3%)

**Table 3:** Risk factors for graft loss in first and second transplantation.

	HR	95% CI	*p* value
Number of transplantations			
1	Ref		
2	1.5	1.1–2.0	0.003
≥3	3.3	1.7–6.1	<0.001

Recipient age (years)			
<50	Ref		
50–59	1.2	0.9–1.6	0.1
60–69	1.9	1.4–2.5	<0.001
≥70	2.9	1.8–4.8	<0.001

Time on dialysis (years)			
<1	Ref		
1-2	1.2	0.9–1.5	0.2
2–4	1.4	1.1–1.8	0.003
>4	1.7	1.3–2.2	<0.001

Charlson–Deyo index^*∗*^			
2 (kidney disease only)	Ref		
3 (≤1 mild comorbidity)	1.2	0.9–1.6	0.1
4 (≥2 mild or 1 moderate comorbidity)	1.8	1.4–2.4	<0.001
≥5 (multiple comorbidities)	2.3	1.7–3.0	<0.001

Smoking	1.3	1.1–1.5	0.01

Comorbidities^*∗*^			
Myocardial infarction	1.3	1.0–1.7	0.04
Congestive heart failure	3.1	2.1–4.5	<0.001
PAD	1.4	1.0–1.8	0.03
Diabetes with end organ failure	1.2	1.0–1.3	0.02
Moderate – severe liver disease	1.3	1.0–1.6	0.04

*Note.* HR, hazard ratio; CI, confidence interval; PAD, peripheral artery disease. ^*∗*^Charlson–Deyo index was analyzed for scoring points and in a second model using the same confounders for underlying comorbidities to avoid interactions.

**Table 4:** Likelihood of retransplantation.

	LR	95% CI	*p* value
Age < 65 years at graft loss	2.7	1.3–5.5	0.008
≤1 light comorbidity (Charlson–Deyo index ≤ 3)	1.5	1.0–2.4	0.04
BMI < 30 kg/m^2^	2.1	1.0–4.8	0.04
Previous graft survival > 5 years	1.5	1.1–2.0	0.01
Prior dialysis < 3 years	1.5	1.2–2.4	0.001
Initial peritoneal dialysis	1.5	1.1–2.2	0.02
Number of previous transplantations	1.4	1.0–2.0	0.04

*Note.* LR, likeliness ratio; CI, confidence interval; BMI, body mass index (kg/m^2^).

**Table 5:** Risk factors for graft survival in retransplantation.

	HR	95% CI	*p* value
Age ≥ 50 years at retransplantation	1.8	1.1–2.9	0.03
≥1 light comorbidity (Charlson–Deyo index ≥ 3)	1.8	1.1–3.0	0.03
Clavien–Dindo index ≥ IV after first transplantation	2.9	1.1–7.5	0.03
Previous graft survival < 2 years	1.6	1.0–2.6	0.04
Prior dialysis ≥ 1 year	1.7	1.0–3.2	0.04
Current smoker	2.6	1.6–4.5	<0.001

*Note.* HR, hazard ratio; CI, confidence interval.

## Data Availability

The data used to support the findings of this study are restricted by the ethics committee in Zürich in order to protect patients' privacy.

## Abbreviations

BMI: Body mass index
CAPD: Continuous ambulatory peritoneal dialysis
DBD: Donor after brain death
DCD: Donor after circulatory death
DSA: Donor specific antibody
ECD: Extended criteria donor
HLA: Human leukocyte antigen
MFI: Mean fluorescence intensity
PAD: Peripheral artery disease.
